# Acute bronchiolitis in infancy as risk factor for wheezing and reduced pulmonary function by seven years in Akershus County, Norway

**DOI:** 10.1186/1471-2431-5-31

**Published:** 2005-08-18

**Authors:** Hans-Olav Fjærli, Teresa Farstad, Gisle Rød, Gunn Kristin Ufert, Pål Gulbrandsen, Britt Nakstad

**Affiliations:** 1 Faculty Division Akershus University Hospital, University of Oslo, 1474 Nordbyhagen, Norway; 2 Department of Paediatrics, Akershus University Hospital, 1478 Lørenskog, Norway; 3 Voksentoppen Centre for Asthma and Allergy in Children, Rikshospitalet, 0791 Oslo, Norway

## Abstract

**Background:**

Acute viral bronchiolitis is one of the most common causes of hospitalisation during infancy in our region with respiratory syncytial virus (RSV) historically being the major causative agent. Many infants with early-life RSV bronchiolitis have sustained bronchial hyperreactivity for many years after hospitalisation and the reasons for this are probably multifactorial. The principal aim of the present study was to investigate if children hospitalised for any acute viral bronchiolitis during infancy in our region, and not only those due to RSV, had more episodes of subsequent wheezing up to age seven years and reduced lung function at that age compared to children not hospitalised for acute bronchiolitis during infancy. A secondary aim was to compare the hospitalised infants with proven RSV bronchiolitis (RS+) to the hospitalised infants with non-RSV bronchiolitis (RS-) according to the same endpoints.

**Methods:**

57 infants hospitalised at least once with acute viral bronchiolitis during two consecutive winter seasons in 1993–1994 were examined at age seven years. An age-matched control group of 64 children, who had not been hospitalised for acute viral bronchiolitis during infancy, were recruited from a local primary school. Epidemiological and clinical data were collected retrospectively from hospital discharge records and through structured clinical interviews and physical examinations at the follow-up visit.

**Results:**

The children hospitalised for bronchiolitis during infancy had decreased lung function, more often wheezing episodes, current medication and follow-up for asthma at age seven years than did the age matched controls. They also had lower average birth weight and more often first order family members with asthma. We did not find significant differences between the RSV+ and RSV- groups.

**Conclusion:**

Children hospitalised for early-life bronchiolitis are susceptible to recurrent wheezing and reduced pulmonary function by seven years compared to age-matched children not hospitalised for early-life bronchiolitis. We propose that prolonged bronchial hyperreactivity could follow early-life RSV negative as well as RSV positive bronchiolitis.

## Background

Acute viral bronchiolitis is one of the most common causes of hospitalisation during infancy in Akershus County, Norway, with respiratory syncytial virus (RSV) historically being the major causative agent. RSV causes respiratory disease in young children worldwide and by the age of two years most children have been infected. In temperate climates the infection occurs as yearly winter epidemics and the impact of RSV on human health is demonstrated annually when infants are admitted to hospitals in large numbers. Symptoms vary from a mild upper respiratory tract infection to severe bronchiolitis with hyperinflated lungs and hypoxemia [1]. The first infection is usually the most severe but milder reinfections are common throughout life. The risk of severe illness is highest in infants born prematurely and in those with chronic lung disease, certain congenital heart defects and immunodeficiency disorders [2,3]. Many infants with severe RSV bronchiolitis experience recurrent wheezing in later childhood and there is growing evidence that early-life RSV bronchiolitis may predispose some infants to the development of childhood asthma [4-6]. The genetic background of the infant, intermittent changes in host cellular immune responses and neural control leading to sustained bronchial hyperreactivity and recurrent wheezing, timing of RSV infection with respect to allergen exposure, environmental conditions and exposure to endotoxin are all factors suggested to contribute to RSV induced asthma [7-9].

Human metapneumovirus (hMPV) is now recognised as one of several other viral pathogens that can cause acute bronchiolitis, the remaining being mainly rhinovirus, parainfluenzavirus, influenzavirus and adenovirus [10]. hMPV have recently been found to be an important cause of acute bronchiolitis in infants and children. However, the long-term effect of this and other viral agents on lung function and symptoms in later childhood is not yet fully investigated [11,12].

The primary aim of this study was to compare the airway symptoms and lung function of children hospitalised for early-life bronchiolitis with age matched controls without such a history at age seven years. We also aimed to describe possible differences between those with proven RSV bronchiolitis and those with non-RSV bronchiolitis regarding the same endpoints.

## Methods

### Study population

During two consecutive years in 1993–1994 a sample of nasopharyngeal aspirate (NPA) was collected from infants hospitalised with acute respiratory disease. The doctors on call conducted, as part of the admission routine, clinical interviews using structured questionnaires and performed physical examination of all infants admitted with suspected acute bronchiolitis.

In the present follow-up study at age seven years hospital records, questionnaires and microbiological results of all children hospitalised during infancy with acute bronchiolitis were analysed in retrospect. 109 infants presenting with respiratory insufficiency such as tachypnoe, intercostal retractions, increased mucus production and soar coughing were eligible for follow-up. However, we excluded 11 infants who were born before completed 37 wGA (weeks of gestational age), 11 infants who had divergent results in two different microbiological tests, 22 infants who had insufficient NPA material for testing, two children who had died from unrelated causes and six children that did not want to participate or had moved far away from our region.

The remaining 57 children were willing and able to take part in the study and came for a follow-up visit at age seven years. Children hospitalised during infancy with verified RSV bronchiolitis (RS+) had to be positive for RSV in two different microbiological tests, and children hospitalised with non-RSV bronchiolitis (RS-) had to be negative for RSV in the same two different microbiological tests.

### Microbiological tests

Two different tests for detection of RSV in NPA were used. These were our own in-house Abbott TestPack RSV enzyme immunoassay (EIA) and immunofluorescens (IF) staining performed in our reference laboratory at the Department of Microbiology, Rikshospitalet, Oslo [13,14].

### Structured clinical interview at the follow-up visit

A clinical interview based on the structured questionnaire used when the children with bronchiolitis were admitted to hospital were performed by three of the authors in the study (H.O.F, T.F., G.R.) to give information on episodes of wheezing (defined by us as episodes of difficult breathing accompanied by a whistling noise in the chest during expiration), number of visits to a family physician, practicing paediatrician and resubmissions to any hospital for wheezing or asthma (as diagnosed by a medical doctor) during the 6–7 years that had passed since the hospitalisation in infancy. Hospital records confirmed number of hospitalisations, whereas the parents gave information regarding visits to a family physician or practicing paediatrician. Information on failure to thrive, regular medications and physical activity was given in the interviews, as was information on parental smoking habits, number of preschool (<6 years old) siblings and prevalence of allergy, eczema and asthma in first order family members (mother, father or siblings) that had been diagnosed by a medical doctor.

### Physical examination and pulmonary function tests at the follow-up visit

The medical doctors who conducted the structured clinical interviews performed weight and height measurements and a general clinical examination including inspection and auscultation at the follow-up visit. All children performed a standard pulmonary function test (PFT) under the supervision and guidance of a skilled paediatric nurse (G.K.U.). Forced vital capacity (FVC), forced expiratory volume in one second (FEV_1_) and forced expiratory flow rate at 50% of FVC (FEF_50_) were measured with the Vmax 20 equipment (SensorMedics). The equipment was calibrated daily according to the specifications of the producer. Measurements of pulmonary function were performed both before and 10 minutes after inhalation of 0,2 mg salbutamol (Airomir Autohaler) and the one attempt (out of three) with the best technical profile both before and after salbutamol was used for analysis.

### The control group

An age-matched control group of seven years old children were recruited from a nearby primary school. A letter was distributed by the school principal to the parents of 92 children (three school classes) providing information about the study and explaining the criteria set for participation. All children had to be healthy term infants born at completed 37 wGA and without history of hospitalisation for respiratory problems during the first year of life. 64 children fulfilled the inclusion criteria, gave informed consents and took part in the study. At the follow-up visit the inclusion criteria were confirmed, an identical structured clinical interview with the parents of the control children were undertaken and the children performed the same PFT as the other children in the study.

### Statistics

For comparison of continuous data between two groups the independent samples T-test was used and for comparison of ordinal and nominal data between two groups we used chi square tests. A p-value <0.05 was considered statistically significant and continuous data were given with 95% confidence intervals (CI). The statistical programme SPSS, version 12.0.1, was used in all calculations.

### Ethics

The regional ethics committee for medical research approved the study and the parents gave oral and written informed consent.

## Results

### Structured clinical interviews

Among the 57 children hospitalised with acute bronchiolitis in infancy, 35 children (61%) tested double RSV positive while 22 (39%) tested double negative for RSV in NPA. Mean birth weight, mean age at hospitalisation and mean length of hospitalisation including admission day were not significantly different between these groups. According to data collected at admission, seven (32%) in the RS- and 15 (43%) in the RS+ group had experienced one or more episodes of a possible lower respiratory tract infection prior to the first hospitalisation for bronchiolitis.

There were not significant differences between the RS- and RS+ group or between these two groups combined and the control group regarding gender, weight or height at the follow-up visit. Mean birth weight was significantly lower in the hospitalised group compared to the control group (tab 1). First order family members of the hospitalised children had significantly higher prevalence of asthma than first order family members of the control group (39% vs 19%, p = 0.015), while no significant differences were observed for other risk factors (tab 1). Within the hospitalised groups no significant differences were found for any of the risk factors (tab 2).

**Table 1:** Follow-up of children hospitalised or not with acute bronchiolitis in infancy

Reported at child age seven years	Hospitalised group				Not hospitalised group	
	RS- (n = 22)	RS+ (n = 35)	95% CI^a^	Both (n = 57)	Controls (n = 64)	95% CI^a^

Age, mean (range) at hospitalisation, months	5.9 (0–11)	5.1 (0–9)	-0.8 2.5			
Length, mean (range) of hospitalisation, days	4.6 (2–20)	4.8 (2–14)	-2.1 1.8			
Birth weight, mean (range), grams^b^	3507 (2420–4300)	3506 (2500–4045)	-225.6 228.5	3506 (2420–4300)	3733 (2100–5220)	-425.2 -27.7
Weight, mean (range) at follow-up visit, kg	27.5 (23–42)	28.3 (22–53)	-4.0 2.4	28.0 (22–53)	29.2 (20–45)	-0.8 3.2
Height, mean (range) at follow-up visit, cm	127.7 (118–139)	127.3 (117–146)	-2.8 3.7	127.4 (117–146)	128.6 (115–145)	-1.0 3.4
Age, mean (range) at follow-up visit, months	93.6 (86–101)	92.8 (86–102)	-1.2 2.9	93.1 (86–102)	94.0 (88–99)	-0.4 2.2
No. boys (%)	12 (54)	20 (57)		32 (56)	36 (56)	

^a ^95% confidence interval (CI) of difference between means

^b ^P = 0.023 with independent samples T-test between the hospitalised and not hospitalised group

**Table 2:** Risk factors in family members of children hospitalised or not with acute bronchiolitis in infancy

Reported at child age seven years	Hospitalised group			Not hospitalised group
No. families with risk factors^a^	RS- (n = 22)	RS+ (n = 35)	Both (n = 57)	Controls (n = 64)

Allergy before seven years	8	13		
Current allergy	12	16	28	33
Eczema before seven years	6	7		
Current eczema	8	14	22	28
Asthma before seven years	10	8		
Current asthma^b^	10	12	22	12
Siblings <6 years before seven years	12	22		
Current siblings <6 years	6	11	17	26
Parental smoking before seven years	10	16		
Current parental smoking	10	19	29	28

^a ^Risk factors in first order family members (mother, father, siblings) as reported by parents

^b ^P = 0.015 with chi square test between the hospitalised group and not hospitalised group

**Figure 1:**
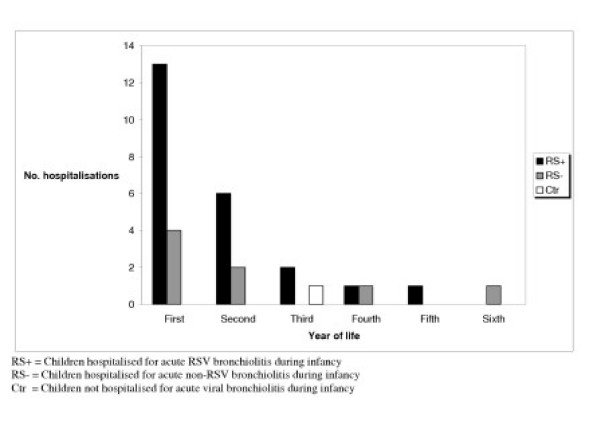
Subsequent readmissions after hospitalisation for acute viral bronchiolitis during infancy.

In the group of children hospitalised with acute bronchiolitis in infancy 32 subsequent hospitalisations in 24 children were noted with peak incidence in the first year of life. Only one child in the control group needed hospitalisation for wheezing during the study period (fig 1).

29 children in the hospitalised group were reported with ≥3 episodes of wheezing during the follow-up period compared to 9 children in the control group (51% vs 14%, p < 0.001). No significant differences were found between the RS+ and RS- groups in this respect (tab 3).

**Table 3:** Wheezing^a ^ever and current asthma in children hospitalised or not with acute bronchiolitis in infancy

Reported at child age seven years	Hospitalised group			Not hospitalised group
	RS- (n = 22)	RS+ (n = 35)	Both (n = 57)	Controls (n = 64)

≥3 episodes of wheezing ever during follow-up^d^	12	17	29	9
Current follow-up for asthma by a medical doctor^b,e^	10	21	31	5
Current medication for asthma^c,e^	8	12	20	5

^a ^Wheezing ever in childhood treated by a family physician or a practicing paediatrician

^b ^Current follow-up for asthma by a family physician or a practicing paediatrician

^c ^Current asthma medication like β-2 agonists or inhaled corticosteroids

^d ^P < 0.001 with chi square test between the hospitalised group and not hospitalised group

^e ^P < 0.001 with chi square test between the hospitalised group and not hospitalised group

At the follow-up visit 31 children in the hospitalised group were currently under supervision for asthma by a practicing paediatrician or a family physician, in contrast to five children in the control group (54% vs 8%, p < 0.001). More children in the hospitalised group than in the control group used asthma medication currently (35% vs 8%, p < 0.01). No statistical differences were found between the RS+ and RS- groups for these parameters (tab 3).

### Pulmonary function tests

No differences were found between the RS+ and RS- groups for FVC, FEV_1 _and FEF_50 _when comparing predicted values and test results before and after inhalation of salbutamol (tab 4). The test results for FVC after salbutamol, FEV_1 _before salbutamol and FEF_50 _before and after salbutamol were significantly worse in the combined hospitalised group than in the control group (tab 4). We did not find differences between groups for reversibility when measured as percent improvement from baseline after inhalation of salbutamol for any of the test parameters (tab 4).

**Table 4:** Spirometry in children hospitalised or not with acute bronchiolitis in infancy

Age seven years	Hospitalised group				Not hospitalised group	
**FVC (liters)**	RS- (n = 22)	RS+ (n = 35)	95% CI^e^	Both (n = 57)	Ctr (n = 64)	95% CI^e^

Predicted^a^	1.78	1.76	-0.12 0.15	1.77	1.82	-0.04 0.14
Before salbutamol	1.65	1.65	-0.17 0.17	1.65	1.75	-0.02 0.19
After salbutamol^c^	1.69	1.69	-0.16 0.17	1.69	1.81	0.004 0.22
Reversibility^b^	2.5	1.9	-2.3 3.6	2.1	3.3	-9.3 3.2
**FEV_1 _(liters)**						
Predicted^a^	1.51	1.50	-0.10 0.12	1.51	1.54	-0.04 0.11
Before salbutamol^c^	1.50	1.51	-0.16 0.13	1.50	1.64	0.05 0.23
After salbutamol	1.57	1.59	-0.16 0.11	1.59	1.70	0.03 0.20
Reversibility^b^	4.9	5.6	-4.0 2.6	5.3	5.6	-3.5 3.9
**FEF_50 _(liters/min)**						
Predicted^a^	2.34	2.33	-0.12 0.14	2.33	2.36	-0.07 0.13
Before salbutamol^d^	1.92	1.99	-0.35 0.20	1.96	2.28	0.15 0.49
After salbutamol^d^	2.17	2.34	-0.43 0.10	2.28	2.56	0.11 0.46
Reversibility^b^	11.7	14.5	-10.0 4.3	13.4	9.6	-9.2 1.6

^a ^According to European Respiratory Society 1993 Update

^b ^Percent improvement 10 minutes after inhalation of 0.2 mg salbutamol

^c ^P < 0.05 with independent samples T-test between the hospitalised group and not hospitalised group

^d ^P < 0.01 with independent samples T-test between the hospitalised group and not hospitalised group

^e ^95% confidence interval (CI) of difference between means

We also performed all analyses excluding the children with prior infection, first from the RS- group, then from both groups. There were not any substantial changes in observations or conclusions. These data are not shown.

## Discussion

The results of this follow-up study show that children hospitalised with acute bronchiolitis during infancy in our region are at increased risk for subsequent childhood wheezing and for reduced pulmonary function, at least up to age seven years. The children were all admitted to the same hospital and from two consecutive winter seasons.

Mean length of hospitalisation including admission day is in accordance with other studies [15]. Mean age at first hospitalisation is higher than in other studies, and 39% of the children had experienced episodes of possible RSV infections before hospitalisation [16,17]. These children were not unevenly distributed on the RS+ and RS- groups. We were not able to observe substantial changes in the results when excluding children with prior infections. This is not surprising. First, since the number of observations is reduced by this procedure, larger differences are necessary to provide significant changes. Secondly, none of these prior infections were severe enough to require admission to hospital. Accordingly, they did probably not meet the criteria of Ruuskanen and Ogra [18] or Court [19], and we assume it rather unlikely that they were due to RSV. Nevertheless, there is a small possibility that we have underestimated differences between the RS negative and positive groups.

We were surprised to find that many of the hospitalised children were deemed RSV negative in NPA, since they were examined in the middle of an RSV epidemic. Obviously, the two microbiological techniques used in our study may not have been sensitive and specific enough due to properties of the tests themselves [20]. In 1993–1994 we did not have access to more sensitive microbiological tests, such as detection of nucleic acid by reverse-transcriptase polymerase chain reaction (PCR) [21]. However, using new knowledge about the performance of the tests we used compared to PCR [22,23], we have calculated that the overall probability of misclassification in our study is 4%, restricted to one or two false negatives. Thus, we find it unlikely that the results in the RSV negative group are contaminated by misclassification of the microbiological agent. For all variables studied, the results in these two groups are so similar, that even a much larger study would not be likely to detect differences.

Several studies have shown that hospitalisation for acute RSV bronchiolitis in infancy is an important risk factor for subsequent wheezing [24,25]. We show that hospitalisation for RSV negative bronchiolitis is an equal important risk factor for subsequent wheezing as is RSV positive bronchiolitis. The intensity of the host inflammatory response, and not only the virus itself, is probably a major determinant to whether or not bronchiolitis develops at the time of infection. This has been well documented in a recent study comparing infants with bronchiolitis due to RSV and influenzavirus [26]. Also, a high frequency of dual infection by RSV and hMPV has been reported in infants with severe bronchiolitis receiving mechanical ventilation [27].

The incidence of childhood asthma, when defined as three or more episodes of wheezing ever during follow-up, was in our study 51% in the hospitalised group and 14% in the control group. Correspondingly, as reported by parents, 54% in the hospitalised group and 8% in the control group were at age seven years consulting for asthma with a family physician or a practicing paediatrician. Our findings further support that hospitalisation for early-life bronchiolitis, not only those due to RSV, is associated with increased prevalence of childhood asthma, at least up to age seven years [28,29].

The percentage of smokers among parents of the hospitalised children in our study was high. However, a high percentage of parents currently smoking was also recorded in the control group. Exposure to tobacco smoke seemed not to be an independent risk factor for childhood wheezing in our study. Several studies have found parental smoking to be an important risk factor for wheezing in early childhood but not in older children. In the Tucson Children's Respiratory Study the greatest impact was found in children under three years of age when mothers were smoking during pregnancy [30].

No statistically significant differences were found between the hospitalised group and the control group in current prevalence of allergy or eczema in first order family members and number of families with preschool siblings. Heredity for asthma in the hospitalised group seemed to be the only significant risk factor in first order family members for childhood wheezing in our study. Several other studies have shown that the prevalence of asthma among family members of infants with acute viral bronchiolitis is slightly higher than in the general population [31].

Early-life RSV bronchiolitis has in many studies been associated with sustained impaired lung function. Our study supports this by demonstrating significantly reduced test results for several parameters in the hospitalised group compared to the control group. Our results are in accordance with a study from Noble et al in which the index children had significantly reduced lung function at nine to ten years of age [32]. According to a study by Sigurs et al severe RSV bronchiolitis in infancy was significantly associated with reduced lung function even at 13 years of age [6].

Studies have shown that pulmonary function at birth of newborns suffering from acute bronchiolitis later on during infancy are reduced compared to pulmonary function at birth of newborns not suffering from acute bronchiolitis later on during infancy [33,34]. This might indicate a congenital pulmonal predisposition for bronchiolitis susceptibility and later obstructive lung disease. A significant lower birth weight in the hospitalised infants in our study might indicate that they had smaller lungs or delayed pulmonal development compared to the controls.

## Conclusion

Acute RSV bronchiolitis is a major health problem in our region with a mean hospitalisation incidence for the period 1993–2000 in infants of 23.6 per 1.000 [35]. Many infants with acute viral bronchiolitis, not only those due to RSV, will have episodes of recurrent wheezing, resubmissions to hospital and reduced lung function, at least up to age seven years. The results of the present study indicate that host factors may be as important as the nature of the infecting agent for the development of bronchiolitis and later childhood wheeze and need further studies.

## Competing interests

The author(s) declare that they have no competing interests.

## Authors' contributions

HOF had primary responsibility for protocol development, outcome assessment, data acquisition and analysis and writing of the manuscript. TF participated in the development of the protocol, outcome assessment, data acquisition and analysis and writing of the manuscript. GR participated in protocol development, data acquisition and analysis and writing of the manuscript. GKU participated in data acquisition and writing of the manuscript. PG participated in data analysis and interpretation and critical revision of the manuscript for important intellectual content. BN participated in outcome assessment, data analysis and writing of the manuscript. All authors have read and approved the final manuscript.

## Pre-publication history

The pre-publication history for this paper can be accessed here:


